# Mobile health in adults with congenital heart disease: current use and future needs

**DOI:** 10.1007/s12471-016-0901-z

**Published:** 2016-09-19

**Authors:** M. J. Schuuring, A. P. Backx, R. Zwart, A. H. Veelenturf, D. Robbers-Visser, M. Groenink, A. Abu-Hanna, N. Bruining, M. P. Schijven, B. J. Mulder, B. J. Bouma

**Affiliations:** 1 Department of Cardiology, Academic Medical Center, Amsterdam, The Netherlands; 2 Department of Cardiology, HAGA Teaching Hospital, the Hague, The Netherlands; 3 Department of Medical Informatics, Academic Medical Center, Amsterdam, The Netherlands; 4 Department of Clinical and Experimental Information processing, Erasmus Medical Center Rotterdam, Rotterdam, The Netherlands; 5 Department of Surgery, Academic Medical Center, Amsterdam, The Netherlands

**Keywords:** Congenital heart disease, GUCH, Mobile health, mHealth, eHealth, Lifestyle, Quality of life, Heart failure, Arrhythmia

## Abstract

**Objective:**

Many adults with congenital heart disease (CHD) are affected lifelong by cardiac events, particularly arrhythmias and heart failure. Despite the care provided, the cardiac event rate remains high. Mobile health (mHealth) brings opportunities to enhance daily monitoring and hence timely response in an attempt to improve outcome. However, it is not known if adults with CHD are currently using mHealth and what type of mHealth they may need in the near future.

**Methods:**

Consecutive adult patients with CHD who visited the outpatient clinic at the Academic Medical Center in Amsterdam were asked to fill out questionnaires. Exclusion criteria for this study were mental impairment or inability to read and write Dutch.

**Results:**

All 118 patients participated (median age 40 (range 18–78) years, 40 % male, 49 % symptomatic) and 92 % owned a smartphone. Whereas only a small minority (14 %) of patients used mHealth, the large majority (75 %) were willing to start. Most patients wanted to use mHealth in order to receive more information on physical health, and advice on progression of symptoms or signs of deterioration. Analyses on age, gender and complexity of defect showed significantly less current smartphone usage at older age, but no difference in interest or preferences in type of mHealth application for the near future.

**Conclusion:**

The relatively young adult CHD population only rarely uses mHealth, but the majority are motivated to start using mHealth. New mHealth initiatives are required in these patients with a chronic condition who need lifelong surveillance in order to reveal if a reduction in morbidity and mortality and improvement in quality of life can be achieved.

## Introduction

Over past decades the life expectancy of children with congenital heart disease (CHD) has increased dramatically, mainly due to the successes of cardiac surgery [1]. At present, nearly all children with CHD can be operated on at young age and more than 95 % reach adulthood. However, many adults with CHD are affected lifelong by cardiac symptoms, reduced quality of life, and cardiac events [2–6]. These events often merit medical therapy, percutaneous interventions, and open-heart surgery to improve survival and quality of life [1, 6–8]. Consequently, adults with CHD are frequently admitted to hospital, entailing high health-related and non-health-related costs to the affected individuals, employers, and to society at large.

Care of adults with CHD is mainly organised at an outpatient clinic. Patients with CHD are usually under lifelong outpatient surveillance. These outpatient visits are brief evaluations of clinical status, patient education and treatment strategies and include an ECG, cardiac imaging and short-term monitoring; such as 24- or 48-hour blood pressure measurements. These outpatient evaluations are only momentary snapshots. The frequency of outpatient visits ranges from a few times a year to once every five years [2]. Continuous monitoring is rare. Consequently, patients may develop symptoms or signs of deterioration between visits, which could therefore be missed.

Mobile health (mHealth) is the provision of medical care facilitated by mobile technologies capable of delivering health information, monitoring clinical signs and enabling direct care and patient education [9]. There are many potential uses of mHealth, such as E‑support, E‑care, tele-monitoring, tele-treatment, teleconsultation and tele-diagnosis [10, 11]; mHealth brings opportunities to stimulate a healthy life style, to remind patients on medication use, and to enhance monitoring in an attempt to improve outcome. However, it is not known if adults with CHD are currently using mHealth or what type of mHealth they will need in the near future.

## Methods

### Study design and population

Consecutive adult patients with CHD who visited the outpatient clinic at the Academic Medical Center in Amsterdam were asked to fill out a questionnaire directly at the outpatient visit. Exclusion criteria for this study were mental impairment and the inability to speak or write Dutch. This is decided by the treating physician. This study was exempted from approval of the Medical Ethics Committee of the Academic Medical Center in Amsterdam (reference number W16_057), because it was not burdensome for the patient. The Ethics Committee gave us permission to link the questionnaire data to electronic medical records.

### Data collection

**Fig. 1:**
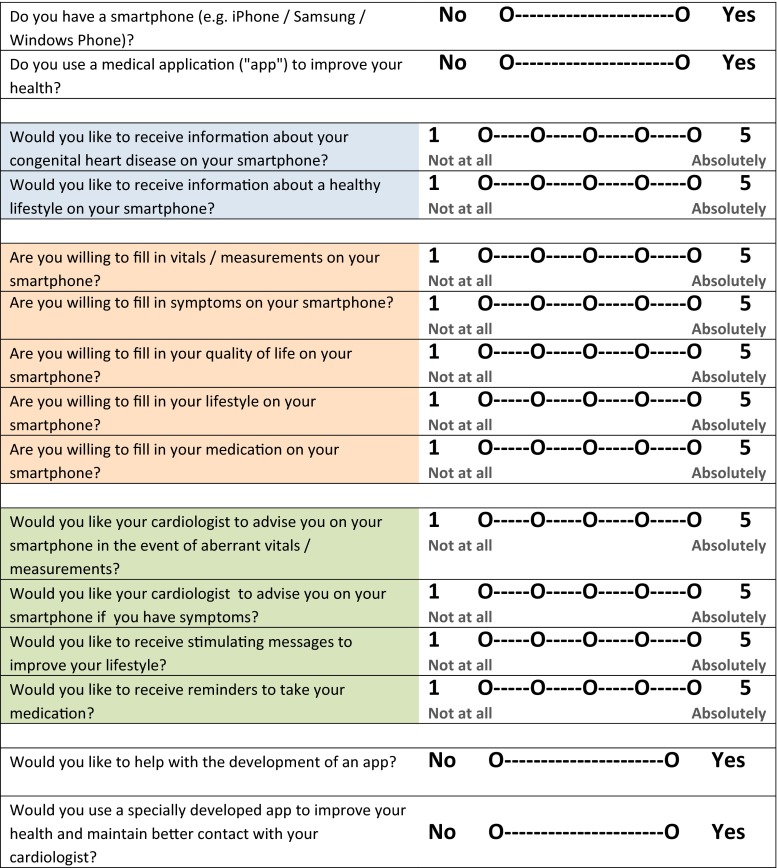
The questionnaire with 15 mobile health-related questions

Data were collected using an mHealth questionnaire, including 15 mobile-health-related questions (Fig. 1). The questionnaire incorporated four general mobile health questions (binary scale) and eleven questions on patient monitoring preferences (Likert scale). These questions on patient preferences included two questions related to information provision, four questions related to willingness of patients to enter data, and four questions related to therapy. In order to make the term mHealth clear to patients and to minimise wide interpretability, the mHealth questionnaire was concentrated on a smartphone. The questionnaire was designed by three authors (MJS, BJM and BJB) and has not been validated. Clinical characteristics were obtained from electronic medical records. Medical records were used to identify cardiac surgery, pacemaker and ICD implantations, use of diuretics and anti-arrhythmic drugs. The type of CHD was categorised as a simple, moderate or complex defect in accordance with the Bethesda conference [12]. Patient functional status was operationalised in accordance with the New York Heart Association (NYHA) classification.

### Statistics

For statistical analysis SPSS 23 (SPSS Inc., Chicago, Illinois) was used. Descriptive statistics for nominal data were presented in percentages. Continuous variables were expressed as mean ± standard deviation when normally distributed, and median if otherwise. Categorical variables were compared with the chi-square test. Independent t‑tests for quantitative data were applied to detect differences between groups. NYHA II, NYHA III and NYHA IV were lumped together in the category symptomatic patients. A *p* ≤0.05 was considered statistically significant.

## Results

### Characteristics of participants

All 118 adults with CHD participated. Median age was 40 (range 18–78) years, 40 % of the patients were male, and 49 % were symptomatic. Table 1 summarises patient characteristics. Amongst participants, 23 % had a simple, 52 % a moderate and 25 % a complex CHD. Most patients had undergone cardiac surgery and antiarrhythmic drugs were prescribed more frequently than diuretics. In total 92 % of all adults with CHD owned a smartphone. The oldest quartile of patients (median age 54 years) used a smartphone significantly less often than the youngest quartile of patients (median age 26 years) (Table 2).

**Table 1:** Clinical characteristics of participating adults with congenital heart disease

	Study group
	*N* = 118
Median age, *years (range)*	40 (18–78)
Male *%*	40
Own a smartphone, %	92
*Disease complexity*	
Simple %	23
Moderate %	52
Complex, %	25
*New York Heart Association*	
Class I %	51
Class ≥ II* %*	49
*Event history*	
Cardiac surgery* %*	76
Pacemaker/ICD implantation %	6
*Medication*	
Diuretics *%*	10
Anti-arrhythmic *%*	30

**Table 2:** Characteristics of younger versus older participating adults with congenital heart disease

	Study group	Study group	*p*
	Youngest quartile	Oldest quartile	
	*N* = 30	*N* = 30	
Use of a smartphone	29	24	*0.026*
Use of a mobile health application	2	3	0.668
Ready to use a mobile health application	21	22	0.781
Ready to help in development of application	15	15	0.436

### Willingness of adults with congenital heart disease to use mHealth

**Fig. 2:**
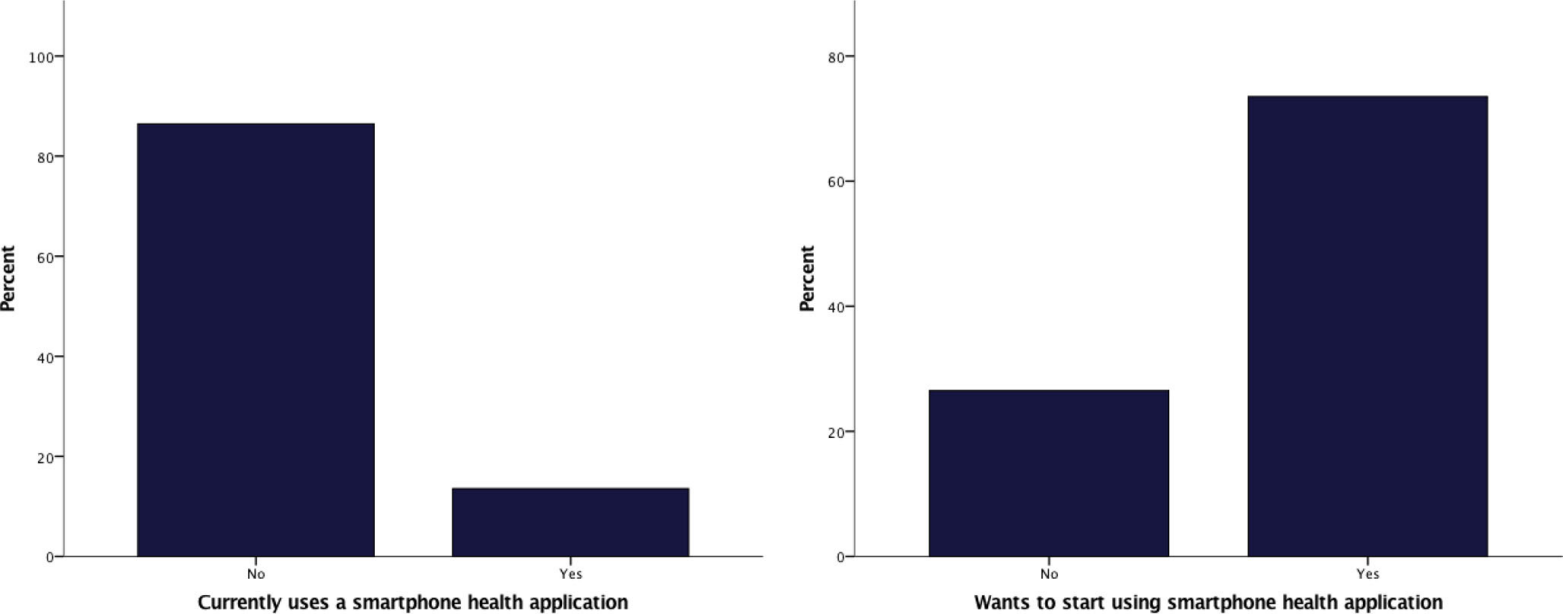
Current mobile health use and willingness to start using mobile health in 118 adults with congenital heart disease

Whereas a small minority (14 %) of patients with CHD already used mHealth, the majority (75 %) were willing to start using mHealth (Fig. 2). There were no differences between the oldest and youngest patient categories in current use of mHealth, willingness to start using mHealth and willingness to help in development of new mHealth initiatives (Table 2).

### Patient preferences on parameters to monitor with mHealth

**Fig. 3:**
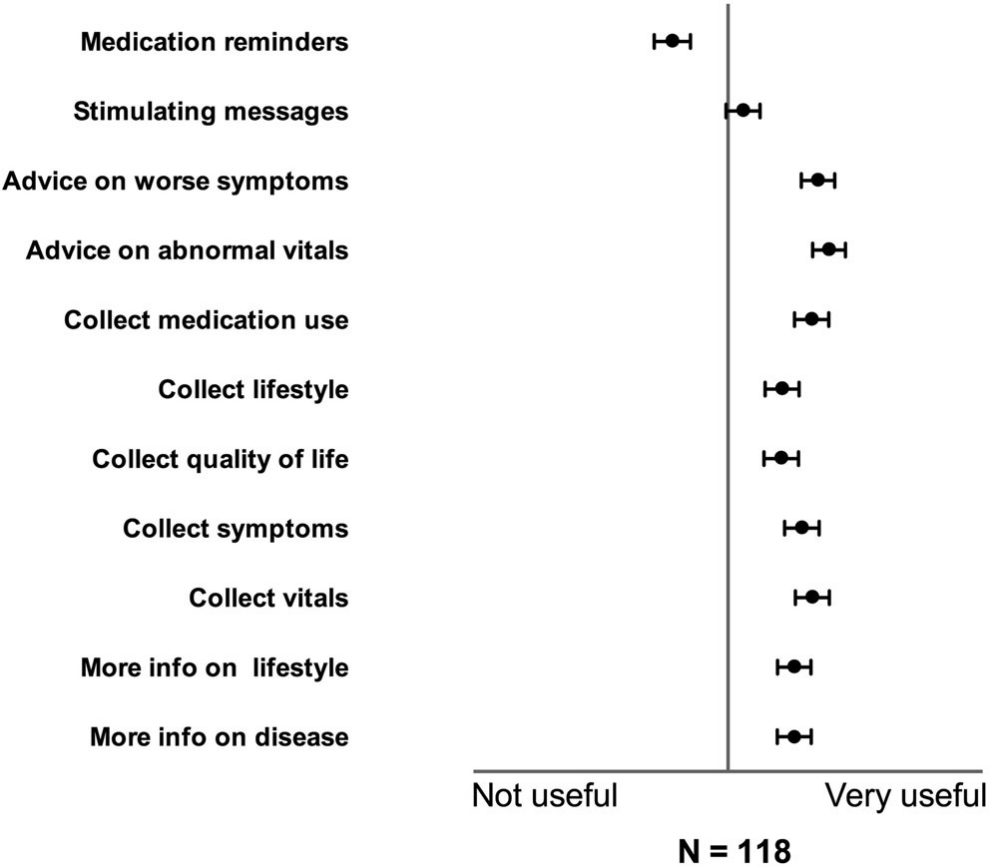
Preferences on parameters to monitor with mobile health in adults with congenital heart disease

The majority of adults with CHD stated willingness to use mHealth to receive information about their disease (71 %), and about a healthy lifestyle (69 %), (Fig. 3). The majority of adults with CHD also stated their willingness to use mHealth to provide information on symptoms (73 %), vitals (75 %) and quality of life (69 %). Therapy, as expressed by advice on worsening symptoms and abnormal vitals, was highly valued (median both 4 out of 5, Fig. 3). The majority of patients wanted to receive advice from their treating cardiologist in the event of aberrant parameters and progression of symptoms, and they also wanted to receive stimulating messages. Medication reminders were not considered useful. No differences in preferences for any particular type of mHealth were found between male and female patients, symptomatic and asymptomatic patients and patients with simple and complex disease (Table 3).

**Table 3:** Subgroup analysis on preferences in mHealth in adults with congenital heart disease

	Male	Female	*p*	Symptomatic	Not symptomatic	*p*	Simple defect	Complex defect	*p*
More info on disease	3.59	3.48	ns	3.51	3.5	ns	3.58	3.63	ns
More info on lifestyle	3.59	3.48	ns	3.36	3.62	ns	3.83	3.37	ns
Collect vitals	3.67	3.66	ns	3.64	3.67	ns	3.83	3.93	ns
Collect symptoms	3.67	3.52	ns	3.58	3.53	ns	3.92	3.74	ns
Collect quality of life	3.43	3.41	ns	3.29	3.47	ns	3.71	3.52	ns
Collect lifestyle	3.38	3.46	ns	3.4	3.39	ns	3.71	3.58	ns
Collect medication use	3.57	3.72	ns	3.64	3.65	ns	4,00	3.63	ns
Advice on abnormal vitals	3.74	3.83	ns	3.69	3.9	ns	4.08	4.22	ns
Advice on worsening symptoms	3.52	3.83	ns	3.7	3.71	ns	3.92	4.08	ns
Stimulating messages	3.07	3.15	ns	3.02	3.16	ns	3.58	3.15	ns
Medication reminders	2.83	2.39	ns	2.29	2.74	ns	2.88	2.44	ns

*ns* not significant

## Discussion

To our knowledge, this is the first report on the readiness of adults with CHD to use mHealth for their condition. The majority of patients with CHD are willing to start using mHealth, but only a small proportion actually uses it currently. All subgroups studied were interested in mHealth, implying that mHealth might be a widely applicable tool in the follow-up of adult patients with CHD.

The adult CHD population is a highly attractive group in which to initiate mHealth initiatives due to their relatively young age, affinity with mobile devices, chronic condition necessitating lifelong surveillance, and the general need to reduce the burden of disease. mHealth has the potential to empower patients and support them in their daily struggles. The additional monitoring of clinical parameters (e. g. heart rate, blood pressure, weight, etc.) might enable physicians and specialised nurses to improve the early recognition of clinical deterioration and to deliver sophisticated patient-tailored care remotely, e. g. titration of diuretics and antiarrhythmic agents. Lifelong surveillance gives clinicians the opportunity to support patients to continue using mHealth. Consequently, mHealth opens opportunities to maintain the motivation to achieve a sustainable improvement. For instance, the short-term beneficial effects of training on exercise capacity in adult patients with CHD have already been demonstrated [13], but without long-term durability when the training period is over [14]. Conceivably, mHealth interventions could overcome this limitation by continuous support and motivational tools.

Overall, studies on the efficacy of mHealth initiatives in cardiology are rare. The results of mHealth studies in heart failure patients, carried out in patients with acquired heart disease, are conflicting [15–17]. Some telemonitoring studies using implantable cardioverter defibrillators have demonstrated that telemonitoring enhances life expectancy and reduces the number of related clinical events in heart failure patients [15]. However, a study using a phone-based telemonitoring system found no differences in all-cause mortality, hospital readmission rates, or readmissions in these patients [17]. Recently, the American Heart Association (AHA) reviewed a total of 13 mHealth studies on prevention of cardiovascular disease and concluded an absence of efficacy data and data on sustainability of engagement by the individual and thus sustainability of the treatment effect, an issue that is extremely important in managing chronic conditions [18]. The European Society of Cardiology is facilitating an action plan pertaining to mHealth issues [9]. This action plan aims at a wider implementation of electronic technology, education and training, in order to play an active role in discussions and to set quality standards. Although adults with CHD are a large group who are particularly suited to mHealth, neither of the position papers comments on this specific patient population.

Both patients and clinicians need to be committed to mHealth interventions in order to achieve long-term impact. In a recent mHealth trial on diet and exercise behaviour in healthy volunteers with an increased risk of atherosclerosis, the dropout rate was as high as 20 % [19]. Therefore, it is important to seek the right balance between time-consuming data collection and dropout. Adults with CHD could benefit from increased adherence because of the necessity for lifelong surveillance [2].

Four other important points are safety, privacy, reimbursement and implementation. At this stage, there is a lack of legal clarity and a lack of transparency regarding the utilisation of the data collected [9]. Data encryption and secured connections are needed to prevent leaks of private data [9]. Before implementation, clear communications on response time are required to prevent patients waiting for a message from a treating physician. For example, outside office hours the telephone number of the cardiac emergency care unit could be shown if immediate attention is necessary, but there would also be the facility to use mHealth to contact a physician with a reasonable response time of 24 h, for instance. There is also significant physician hesitation about implementing mHealth. Patients could potentially overload physicians and nurses with additional work and medical professionals have concerns about the quality of the data generated by mHealth devices [11, 20]. Additionally, many physicians are not reimbursed for mHealth. At this stage mHealth is only reimbursed in a limited number of cases, and reimbursement is commonly not in proportion to the time required [11].

Our study has several limitations. At first, the mHealth questionnaire was confined to a smartphone in order to minimise vagueness about the term mHealth. However, there are many other forms of mHealth. Secondly, the questionnaire was designed by three authors and was not validated.

## Conclusion

The adult CHD population rarely uses mHealth, but the majority is motivated to start using mHealth for their condition. These patients seem particularly attractive for new mHealth initiatives because of their young age, affinity with mobile devices, chronic condition with the necessity of lifelong surveillance, and the general need to reduce the burden of disease. New mHealth initiatives are needed to reveal whether a reduction in morbidity and mortality and improvement in quality of life can be achieved with early event recognition and intervention.
